# A Case Report and Literature Review of Pseudo-TORCH Syndrome Type 2 (PTORCH2)

**DOI:** 10.1155/2022/3555532

**Published:** 2022-10-22

**Authors:** Rami A. Misk, Lama Qawasme, Fawzy M. Abunejma, Bahaa Ibrahim Abu Rahma, Ehab Mohammad Abuawwad, Raja Imad Abu Iram, Abdulrahman Hussein Karaki, Tareq Z. Alzughayyar, Jihad Samer Zalloum

**Affiliations:** ^1^College of Medicine and Health Sciences, Palestine Polytechnic University, Hebron, State of Palestine; ^2^Department of Plastic and Reconstructive Surgery, Hamad General Hospital, Hamad Medical Corporation, Doha, Qatar; ^3^Faculty of Medicine, Al-Quds University, Jerusalem, State of Palestine; ^4^Palestinian Red Crescent Hospital, Hebron, State of Palestine

## Abstract

A pseudo-TORCH syndrome is a rare autosomal recessive disease characterized by intracranial calcification and microcephaly, leading to spasticity and seizures, but the serology of TORCH infection is negative. We present a 4-day-old female patient with jaundice, abnormal movement, and convulsions who was found to be homozygous for the missense *USP18* gene mutation that causes pseudo-TORCH syndrome 2 (PTORCH2). The patient was managed with conservative measures.

## 1. Introduction

Pseudo-TORCH syndrome (PTS), also known as band-like calcification with simplified gyration and polymicrogyria (BLCPMG), is an autosomal recessive condition that often appears with intracranial calcification and polymicrogyria, hepatosplenomegaly, pathology in the white matter, enlarged ventricles, microcephaly, thrombocytopenia, and aberrant neurological behavior in the neonate [1]. However, this clinical presentation resembles the intrauterine TORCH infection in clinical and neuroradiological features but in the absence of serological evidence, and from this point comes the name “pseudo-TORCH.”

Another autosomal recessive condition that clinically mimics pseudo-TORCH syndrome is Aicardi–Goutieres syndrome (AGS), which is caused by harmful mutations in at least seven genes involved in encoding enzymes involved in the intracellular metabolism of nucleic acids, and these genes are as follows: *IFIH1, RNASEH2C, RNASEH2B, RNASEH2A, ADAR, TREX1, or SAMHD1* [2, 3]. These defects activate the response of interferon (IFN) type 1, which causes Aicardi–Goutieres syndrome, which causes PTS. Depending on the defective gene, there are three types of the pseudo-TORCH syndrome [4].

In this study, we are reporting a 4-day-old female patient who was shown to be homozygous for a missense variant in the *USP18* gene, which causes pseudo-TORCH syndrome 2 (PTORCH2).

## 2. Case Presentation

This female patient is the child of a first-degree cousin of a Palestinian couple. She was delivered at 38 weeks of gestation by vaginal delivery in good condition with an Apgar score of 8 at 1 minute and 9 at 5 minutes. On examination in the nursery, we noticed that she had a small head circumference (HC) of 30 cm. She passed meconium in the first 24 hours, fed well, and was discharged after 7 hours against medical advice. There were no immediate postnatal problems.

The patient presented at the age of 4 days, complaining of yellowish discoloration of the skin and sclera. She was admitted for further investigation.

On the first admission at the age of 4 days, she was afebrile, ill, pale-looking, and greenly jaundiced. The examination of her fundi was normal; she was active and had good reflexes, power, and tone. On heart examination, there was a grade 2 continuous murmur, and the echocardiogram showed a tiny patent ductus arteriosus; however, the echocardiogram was repeated at the age of 18 days, which showed a closed duct. Abdominal examination showed a palpable liver 4.5 cm below the right costal margin with a round border and firm consistency. Liver enzymes and direct bilirubin were elevated (total bilirubin = 12.7 mg/dl) at the age of 9 days, with a gradual decrease in bilirubin over the months (total bilirubin = 0.9 mg/dl) at the age of 2 months, and remained in this range. Also, we found that the patient suffered from thrombocytopenia, anemia, and microcephaly.

The patient developed respiratory distress in the form of tachypnea, desaturation, and cyanosis a day after admission, so they started on O_2_ therapy by nasal cannula. At age 15 days, she developed another episode of respiratory distress and needed intubation for 9 days, and at age 4 months, she developed right-sided pneumonia, effusion, and respiratory failure.

**Figure 1:**
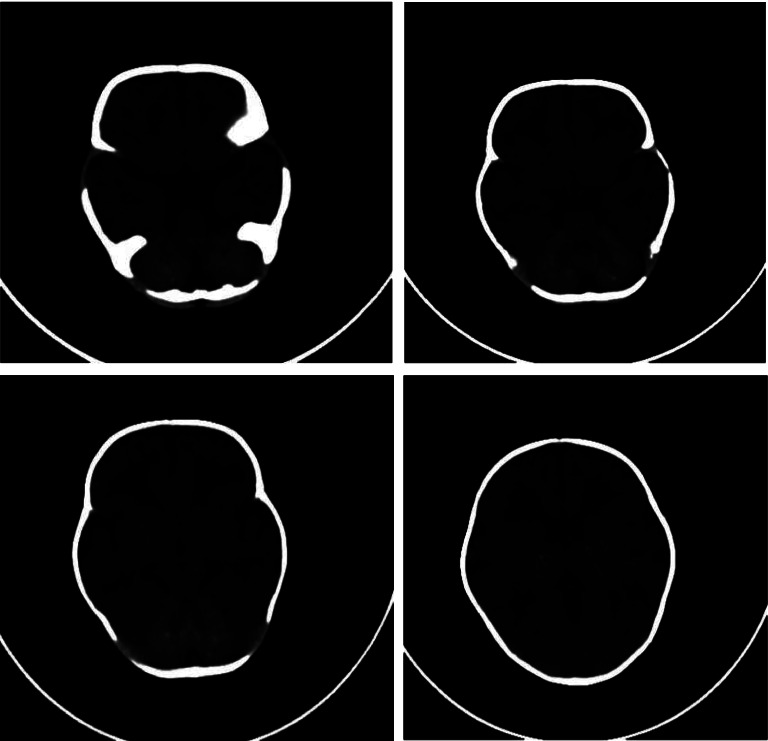
Brain CT scan: diffuse calcifications involving both lobes as well as basal ganglia and extensive bilateral periventricular hypodensities.

At the age of 18 days, the patient was observed to have abnormal movement, including facial twitching and a generalized myoclonic seizure. A cerebral function monitor (CFM) and EEG were done and showed a picture of the suspiciously abnormal electrical activity of the brain. Phenobarbital was administered for one month and then discontinued with no recurrence of seizures. Trans-fontanel ultrasound (TFU) was performed and showed hyperechoic basal ganglia bilaterally. A brain CT scan was done and showed diffuse calcifications involving both lobes as well as basal ganglia and extensive bilateral periventricular hypodensities [Figure 1]. An MRI was not done.

At age 3 months, she developed hepatosplenomegaly and esophageal varices as a result of portal hypertension that ended up resulting in upper GI bleeding. CSF analysis was performed and revealed no abnormalities or growth in cultures at any time. Hematological indices showed anemia and thrombocytopenia, which was due to frequent blood sampling and hepatosplenomegaly, so she needed packed RBCs and platelet transfusions six times each.

As a result of her findings and lab results, we had to put TORCH infection at the top of our differential diagnosis, and a TORCH study was done and showed a negative result. Urine CMV-PCR, HCV, HBV, and HIV screening were also done and showed negative results for both the baby and the mother. The pseudo-TORCH syndrome was considered and exome sequencing was performed. The result was a homozygous missense mutation in the *USP18* gene.  Table 1 displays the results of single exome sequencing analysis.

At the age of 4 months, she was admitted as a case of pseudo-TORCH syndrome 2 (PTORCH2), right-sided pneumonia, bilateral pleural effusions, pulmonary hypertension, upper GI bleeding, respiratory failure, thrombocytopenia, and anemia. Her situation was deteriorating, and she developed severe O_2_ desaturation with hypotension. Then she started to have bradycardia. CPR was done for 15 minutes without improvement, and unfortunately, she was pronounced dead.

## 3. Discussion

In a comprehensive compilation and analysis of the literature and data gathered from multiple pseudo-TORCH syndrome-related studies, Table 2 reviews 14 reported cases of pseudo-TORCH syndrome patients, all of them less than 10 years old. From these 14 patients—7 females and 5 males—the average age at diagnosis was 1.2 years. Except for two patients, the majority of the patients have a clean family history. One of them has a family history of multiple hand malformations, and the other one has a brother with the pseudo-TORCH syndrome. Consanguinity is found in 50% of cases, which is significantly notable. The most common findings in patients are intracranial calcifications and dysmorphic features, which occur in 100% of cases, followed by seizures in 13 of 14 (93%), and microcephaly in 83% (10 of 12). For another 2 patients, information was not available. Finally, less common findings like Congenital thrombocytopenia, which was found in 4 patients, and hepatosplenomegaly, which was found in 2 patients, were also mentioned. Based on the literature, it is a very rare disease. Unfortunately, due to the rarity of the disease, we are unable to provide more information about the best approach to treating those patients (Table 2).

PTS is a group of heterogeneous clinical disease entities that have autosomal recessive inheritance patterns with clinicoradiologically suggestive features that resemble the congenital TORCH infection. They are characterized by the presence of intracranial calcifications, thrombocytopenia, microcephaly, and seizures. However, these manifestations may be due to genetic or nongenetic conditions. The most common cause of nongenetic conditions that cause these manifestations is TORCH infection. However, in the case of a pseudo-TORCH syndrome, all confirmatory tests in search of infectious agents are negative. [5, 7].

So, children are considered to have pseudo-TORCH syndrome if they have the clinical phenotype that suggests in utero exposure to infections, but the disorder has a noninfectious etiology. The high frequency of consanguinity among families with PTS suggests that many of these cases have a genetic basis and are inherited as autosomal recessive traits. In accordance with this, as we mentioned before, the parents of our patient were first cousins [8–10].

One of the notable manifestations in our patient was respiratory involvement, which was noted in several reported cases. The respiratory involvement was significant, leading to respiratory failure in some cases and respiratory arrest in others [5, 6, 8].

The calcifications, which are the most important radiological landmark in PTS, could be both intracranial and extracranial calcifications. The differential diagnosis of intracranial calcifications in an infant includes intrauterine congenital infections (TORCH group), which have been described in association with toxoplasmosis, rubella, cytomegalovirus, herpes simplex, and parvovirus. On the other hand, these calcifications may be due to genetic noninfectious conditions, such as Aicardi–Goutieres syndrome (AGS), Sturge–Weber syndrome (SWS), and tuberous sclerosis. Calcifications in such genetic conditions are typically diffuse cerebral rather than periventricular. In the case of SWS, the calcifications are absent or minimal in neonates and infants. However, in tuberous sclerosis, there are subependymal nodules that were absent in our patient [7, 11, 12].

Aicardi–Goutieres syndrome (AGS) is an important differential of noninfectious etiology and is characterized by progressive neurological dysfunction, acquired microcephaly, CSF lymphocytosis, elevated CSF neopterins and biopterins, and chilblain-like skin lesions. However, these features were absent in our patient, making the diagnosis of AGS unlikely [12, 13]. As a result, the pseudo-TORCH syndrome should be suspected due to the absence of features that suggest other similar conditions and the presence of characteristics of band-like intracranial calcifications.

The pseudo-TORCH syndrome is an umbrella term composed of several syndromes caused by different genetic defects and pathological mechanisms. Depending on the defective gene, there are three types of pseudo-TORCH syndrome. The most common mimicker of PTS is AGS, which is accepted as an interferonopathy [11, 14, 15].

In recent years, a new interferonopathy has been described to cause PTS: human ubiquitin-specific peptidase 18 (*USP18*) deficiencies. *USP18* is a key negative regulator of type I IFN signaling, and its deficiency represents the genetic disorder of PTS and specifically pseudo-TORCH syndrome 2 (PTORCH2) that is caused by dysregulation of the response to type I IFN, which is represented by early-onset intracranial calcifications, thrombocytopenia, and other features of the pseudo-TORCH phenotype [8, 11].

AGS and other interferonopathies can be distinguished from inherited genetic PTC by the presence of a high level of interferon-alpha (INF) in the CSF. We could not evaluate the IFN levels in the CSF. However, CSF analysis did not show lymphocytosis, which reduced the likelihood of such a diagnosis [16–19].

The other two forms of PTS are pseudo-TORCH syndrome-1 (PTORCH-1) and pseudo-TORCH syndrome-3 (PTORCH3). Pseudo-TORCH syndrome -1 (PTORCH-1) is an inherited autosomal recessive disorder characterized by early-onset infantile seizures, developmental delay, spasticity, microcephaly, and intracranial calcifications. PTORCH-1 is caused by mutations in the gene encoding occludin (*OCLN*). Pseudo-TORCH syndrome-3 (PTORCH3) is an autosomal recessive disorder characterized by immune dysregulation and neuroinflammation. Affected infants have developmental delays with acute episodes of fever and multisystem organ involvement. PTORCH-3 is caused by sequence variations in the *STAT2* gene (Signal Transducer and Activator of Transcription 2) [20, 21].

Our case was significant since it brought attention to a very rare condition and created awareness about the early diagnosis of this genetic disorder, which mimics TORCH. Identification of the gene(s) involved in this disorder will provide a unique opportunity to understand the underlying mechanisms. Further genetic studies are needed to develop a specific treatment that targets this mutated gene.

## Figures and Tables

**Table 1:** Result of single exome sequencing analysis.

Gene	Position	Nucleotide
USP18	22 : 18655936	c.911T > A
Amino acid	Zygosity	ACMG classification
p.Leu304His	Homozygous	PM5

**Table 2:** Review of the literature.

First author and publication year	Vivarelli 2001	Vivarelli 2001	Vivarelli 2001	Vivarelli 2001	Kulkarni 2010	Ekinci 2020	Briggs 2008	Briggs 2008	Knoblauch 2003	Knoblauch 2003	Abdel‐Salam 2008	Abdel‐Salam 2008	Patnaik 2017
Sex	F	F	F	F	M	F	M	F	M	M	F	M	M
Age	3 y	20 m	2 y	9 y	NA	11 m	1 w	1 d	Antenatal	Antenatal	39 w	Died at 8 d	8 m
Birth weight (kg)	2.4	2.2	3.2	2.1	2.5	2.5	3.72	3.39	2.3	1.8	NA	1.35	NA
Mother age	30 y	NA	NA	NA	26 y	NA	NA	NA	NA	NA	17	NA	NA
Gestational age	39 w	39 w	NA	35 w	36 w	38 w	38 w	38 w	37 w	32 w	39 w	32 w	NA
Mode of delivery	NVD	NVD	NVD	NVD	NVD	NA	NVD	CS	CS	CS	NVD	NVD	NA
Pregnancy outcomes	Recurrent threatened miscarriages after a week	Free	Repeated threatened miscarriages 2^nd^ after 2 month	Meckel diverticulum perforation and jejunal atresia	At 28 neonate had intracranial calcifications	Free	Free	Seizures	Free	Preterm birth	Free	Free	NA
Past medical history of the mother	Free	Free	Free	Free	Free	NA	NA	NA	NA	NA	Free	Free	NA
Family history	NA	NA	NA	Multiple hand malformations	Free	NA	Free	Pseudo-TORCH (brother)	Free	Free	Free	Free	NA
Apgar score	8	8	8	9	7 at 1 min/9 at 5 min	NA	9 at 5 min	9 at 1 min and 9 at 10 min	9 at 10 min	7 at 10 min	NA	NA	Insignificant
Consanguinity	−	−	+	+	+	+	−	+	−	−	+	+	NA
Intracranial calcifications	+	+	+	+	+	+	+	+	+	+	+	+	+
Microcephaly	+	+	+	+	+	+	−	−	NA	NA	+	+	+
Seizures	+	+	+	+	−	+	+	+	+	+	+	+	+
Congenital thrombocytopenia	−	−	+	+	−	NA	−	−	+	+	NA	NA	NA
Congenital hepatosplenomegaly	NA	NA	NA	NA	−	NA	−	−	+	+	−	−	NA
Dysmorphic features	+	+	+	+	+	NA	+	+	+	+	+	+	NA

NA: nonavailable; NVD: normal vaginal delivery; CS: caesarian section.

## Data Availability

The data used to support the findings of this study are available from the corresponding author upon request.
